# PSMA-Targeted Therapy: Advancements in Detection and Treatment Modalities with Dr. Scott T. Tagawa

**DOI:** 10.3390/cancers16101833

**Published:** 2024-05-11

**Authors:** Viviana Cortiana, Jade Gambill, Harshal Chorya, Diksha Mahendru, Fabiha Amin, Chandler H. Park, Yan Leyfman

**Affiliations:** 1 Department of Medical and Surgical Sciences (DIMEC), University of Bologna, 40126 Bologna, Italy; 2 Parker University, Dallas, TX 75229, USA; 3 Medical College Baroda, Vadodara 390018, India; 4 Global Remote Research Scholars Program, St Paul, MN 55101, USA; 5 Valley Stream South High School, Valley Stream, NY 11581, USA; 6 Norton Cancer Institute, Louisville, KY 40202, USA; chandler.park@louisville.edu; 7 Icahn School of Medicine at Mount Sinai, Oceanside, NY 11572, USA; yan.leyfman@mssm.edu

**Keywords:** prostate cancer, prostate-specific membrane antigen (PSMA), PET, prostate cancer metastasis, prostate cancer diagnosis, prostate cancer treatment

## Abstract

**Simple Summary:**

Prostate cancer presents significant challenges due to its high incidence and prevalence, as it is the most common non-skin cancer in men. The timely detection of prostate cancer and its metastasis is crucial for patient outcomes. Prostate-specific membrane antigen (PSMA) emerges as a promising biomarker for its early detection, due to its specificity and membrane localization on tumor cells. Utilizing PSMA-targeting particles in conjunction with positron emission tomography (PET) scans enhances the accuracy of tumor detection compared to PET alone. This advancement has led to innovative treatment modalities such as Prostate-specific membrane antigen-targeted radionuclide therapies (PSMA-TRTs), which have shown promise in reducing or eliminating tumors, as evidenced by declines in prostate-specific antigen (PSA) levels post treatment. However, PSMA-TRT carries both benefits and adverse effects, with the long-term ones as yet unknown. The short-term adverse effects include fatigue, nausea, pain flares, and potential radiation exposure to others. Further research is needed to explore PSMA-TRT’s long-term efficacy and potential applications beyond prostate cancer.

**Abstract:**

Prostate cancer is one of the most challenging malignancies due to its high incidence and prevalence, as it is the most frequently diagnosed non-skin cancer in men. The timely identification of prostate cancer and its metastasis is paramount for ensuring favorable outcomes for patients. Prostate-specific membrane antigen (PSMA) emerges as a promising biomarker for its detection, due to its specificity. This makes it an ideal target for the early identification of a metastatic phenotype. Situated on the membrane of tumor cells, PSMA facilitates the attachment of PSMA-targeting particles, enabling their detection through positron emission tomography (PET) scans with relative ease. Utilizing these imaging agents in conjunction with PET scans enhances the accuracy of prostate cancer tumor detection compared to PET scans alone. The advancement in prostate cancer imaging has paved the way for innovative treatment modalities. Prostate-specific membrane antigen-targeted radionuclide therapies (PSMA-TRT) exploit PSMA imaging agents to target identified prostate cancer malignancies with precise radiation, thereby reducing or eliminating the tumor mass. PSMA-TRT exhibits significant promise in prostate cancer therapy, evident from the notable declines in prostate-specific antigen (PSA) levels post treatment. However, PSMA-TRT carries both beneficial and adverse effects. While it represents a substantial leap forward in tumor cell imaging, PSMA-based antigens, being larger particles than ligands, offer prolonged imaging capabilities. Yet, the long-term effects of PSMA-TRT remain unknown, with the short-term adverse ones including fatigue, nausea, pain flares, and potential radiation exposure to others.

## 1. Introduction

Prostate cancer poses a significant health challenge globally, with approximately 1.4 million new cases diagnosed worldwide in 2020 alone [1]. It stands as the most common non-skin cancer among men in the United States, ranking as the second leading cause of cancer-related mortality in this population [2]. This prevalence highlights the critical need for effective treatment strategies, particularly for advanced disease stages. Advanced prostate cancer, often characterized by the spread of cancer cells beyond the prostate gland, presents significant challenges during its management and requires innovative approaches to improve its outcomes. Despite advancements in its treatment, the complexity and variability of prostate cancer requires the continued innovation of therapeutic strategies. One promising approach is the targeting of the prostate-specific membrane antigen (PSMA), a protein highly expressed on the surface of prostate cancer cells [3]. The prostate-specific membrane antigen (PSMA), a type II transmembrane glycoprotein originally characterized by the murine monoclonal antibody (mAb) 7E11-C5.3, is expressed in all forms of prostate tissue, including carcinoma, and functions as a folate hydrolase and N-acetylated-alpha-linked-acidic dipeptidase (NAALADase), playing crucial roles in glutamate production and folate absorption [4]. This commentary explores the transformative potential of PSMA-targeted therapy in revolutionizing care for patients with prostate cancer, inspired by the MedNews Week Keynote Conference from Dr. Scott T. Tagawa, which was given on 24 May 2023 [5].

PSMA-targeted therapy offers a precise and targeted approach to treating prostate cancer [3]. By delivering high doses of drugs or radionuclides directly to PSMA-expressing cancer cells, this approach minimizes the damage to surrounding healthy tissues, potentially reducing side effects and improving treatment outcomes. The versatility of PSMA-targeted therapy is further underlined by its potential for combination with other treatment modalities, such as chemotherapy, radiosensitizers, immunotherapy, and PSMA-targeted nanomedicines, suggesting a multifaceted strategy to combat prostate cancer [6].

The ongoing clinical trials and research efforts highlighted in this commentary aim to optimize PSMA-targeted therapy. These studies not only evaluate the efficacy and safety of PSMA-targeting agents but also explore novel combinations and dosing strategies to maximize the therapeutic benefits. Moreover, the emphasis on personalized treatment approaches based on PSMA expression levels reflects a shift towards tailored therapies that consider individual variations in cancer biology and stage [7].

Beyond its impact on prostate cancer treatment, PSMA-targeted therapy holds promise for broader applications in oncology. However, it is important to acknowledge that PSMA is also expressed in non-malignant tissues, which presents a potential limitation that we should be aware of. The expression of PSMA on the immunovasculature of other solid tumors suggests this therapy’s potential application to various cancer types, expanding the scope of targeted cancer therapy.

**Figure 1:**
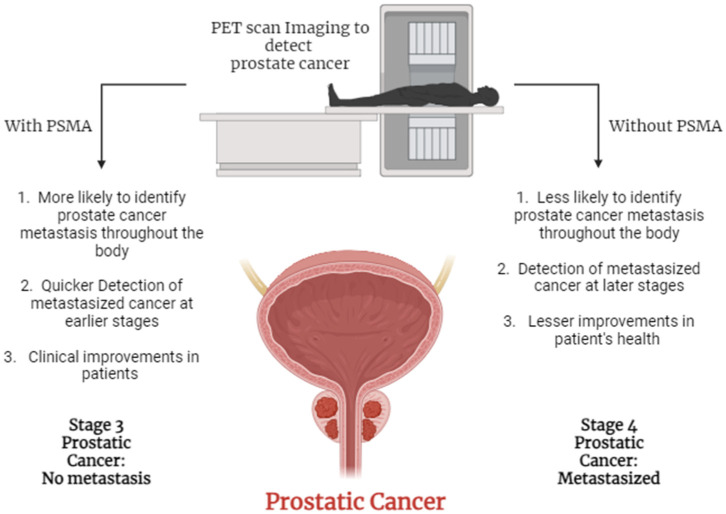
The figure illustrates the enhanced effectiveness of PET scan imaging with PSMA compared to without PSMA. PSMA-enhanced images offer a clearer and more precise detection of prostate cancer lesions, aiding in early detection and treatment decision-making. Created with www.BioRender.com (accessed on 29 March 2024).

In conclusion, PSMA-targeted therapy represents a significant innovation in the management of prostate cancer, offering a targeted and personalized approach to treatment. This commentary aims to present and discuss the key insights and implications of PSMA-targeted therapy, offering a critical analysis of its potential in transforming care for patients with prostate cancer (Figure 1).

## 2. The Role of Prostate-Specific Membrane Antigen (PSMA) in Precision Diagnosis and the Targeted Therapy of Prostate Cancer

As highlighted by Dr. Scott T. Tagawa, prostate-specific membrane antigen (PSMA) is a pivotal protein found in prostate tissue, including carcinoma, originally identified using the murine monoclonal antibody (mAb) 7E11-C5.3 [8]. Its structure is unique, consisting of a 19-amino-acid internal portion, a 24-amino-acid transmembrane portion, and a 707-amino-acid external portion. Interestingly, the PSMA gene is situated on chromosome 11’s short arm, a region rarely deleted in prostate cancer, emphasizing its significance in the disease [4]. Prostate-specific membrane antigen (PSMA) has emerged as a pivotal player in both the diagnosis and treatment of prostate cancer. While PSMA’s role extends beyond the prostate tissue, its widespread investigation and validation in both laboratory and clinical settings underscore its significance. Specifically, PSMA has become the focal point of diagnostic imaging for prostate cancer, particularly through PET imaging, and holds promise as a target for therapy. It is worth noting that while PSMA is a prominent target, other pathways, such as the androgen receptor, also play crucial roles in therapeutic interventions [9,10]. Its prostate-restricted nature means that it is predominantly found in prostate and prostate cancer cells, making it a relatively more precise target for therapies with often balanced collateral damage to healthy tissues. In line with its growing importance, the (joint) EJNMMI/SNMMI guidelines on PSMA PET and therapy provide comprehensive recommendations for the use of PSMA-based imaging and therapy in clinical practice, further solidifying its role in the management of prostate cancer. The (joint) EJNMMI/SNMMI guidelines on PSMA PET and therapy serve as a valuable resource for clinicians, researchers, and policymakers involved in the management of prostate cancer. By promoting standardized approaches to PSMA-based imaging and therapy, these guidelines aim to improve patient outcomes, enhance clinical decision-making, and advance the field of precision oncology. PSMA’s location on the cell membrane, unlike PSA, which is produced inside the cell, makes it particularly intriguing. This distinction allows for the highly specific targeting of prostate cancer cells, offering a pathway to develop treatments that can selectively act on cancerous cells while sparing healthy ones. Beyond prostate cancer, PSMA has also been found in other tumors, expanding its potential applications across different cancer types [11].

The specificity of PSMA has paved the way for novel imaging and treatment strategies. By attaching imaging agents or therapeutic drugs to PSMA-targeting particles, researchers and clinicians can visualize prostate cancer cells using techniques like positron emission tomography (PET) [12]. This targeted approach not only enables precise diagnosis but also facilitates the delivery of therapeutic agents directly to cancer cells, potentially enhancing treatment efficacy and reducing side effects.

Over the last decade, PSMA-targeted imaging agents have undergone remarkable advancements, particularly in their application for the diagnosis and staging of prostate cancer (PCa), including its recurrence. The initial application of PSMA as a PET tracer for PCa marked a significant turning point, sparking widespread recognition of its potential. Despite encountering initial hurdles, continuous research endeavors have propelled the development of increasingly effective agents, and notably the emergence of 18F- and 68Ga-based PSMA-targeted PET imaging agents [13]. These newer agents have demonstrated their superior sensitivity and specificity in detecting lesions associated with prostate cancer, revolutionizing diagnostic accuracy and providing invaluable insights for treatment planning and monitoring. Today, these agents have firmly established themselves as integral components of the standard care protocols in the United States, reflecting their pivotal role in clinical practice. By tracing their trajectory from early breakthroughs to their current prominence, it becomes evident that PSMA PET imaging has evolved into an indispensable tool for diagnosing and staging prostate cancer, particularly in cases of recurrence, offering clinicians unprecedented precision and confidence in patient management.

Beyond diagnosis, PSMA-targeted imaging plays a crucial role in aiding treatment decisions, especially in identifying metastatic lesions and guiding metastasis-directed therapy. This targeted approach has the potential to improve patient outcomes by allowing clinicians to tailor treatments based on the specific characteristics of the disease.

Therefore, PSMA represents an invaluable resource in the overall management of prostate cancer. Its unique characteristics make it an ideal target for precise imaging and precision therapy, offering new hope for patients and clinicians. Continued research and innovation in PSMA-targeted approaches are therefore expected to further refine prostate cancer management and improve patient outcomes.

## 3. PSMA in Prostate Cancer Treatment: Innovations in Radiation Therapy and Targeted Therapy

Dr. Scott T. Tagawa thoroughly examined the current and forthcoming advancements in prostate cancer treatment, with a particular emphasis on the important role of prostate-specific membrane antigen (PSMA) in synergy with radiation therapy [14]. Radiation therapy remains, indeed, a foundational component of PCa treatment, serving both curative and palliative purposes [15]. Its therapeutic modalities involve external beam techniques such as Intensity-Modulated Radiation Therapy (IMRT) and brachytherapy, including the application of encapsulated radioisotopes. There is a significant dose–response relationship; therefore, repopulation as a mechanism of resistance occurs as a not negligible challenge.

A significant advancement in the field is the emergence of PSMA-targeted radionuclide therapy (PSMA-TRT). This innovative approach involves administering systemic radiation that is precisely directed toward PSMA-positive cells [16]. Key radioligands like 177Lu-PSMA-617 were spotlighted, showcasing their efficacy and toxicity profiles, which are linked to their distinctive properties. PSMA-targeting vehicles were also discussed, with a focus on their possible variations in size and their impact on kinetics and biodistribution. There are currently several ongoing trials which highlight the promising results of PSMA-TRT in patients who have undergone various conventional treatments. Interestingly, patients showcased remarkable declines in PSA levels and clinical improvements following PSMA-TRT.

Furthermore, a comprehensive analysis was presented regarding the implications of PSMA-targeted radionuclide therapy (PSMA-TRT) across several demographic groups, considering factors such as age, ethnicity, and comorbidities. The safety profiles and observed side effects were then examined to better understand their potential impact on patient outcomes and quality of life, which is unfortunately not negligible. Moreover, the discussion extended to the topic of re-treatment with PSMA-TRT, suggesting possible strategies for managing disease progression and treatment resistance over time. In this matter, PSMA imaging during treatment planning remains fundamental, given its role in not only accurately diagnosing prostate cancer but also in monitoring treatment responses and guiding personalized therapeutic strategies. This leads to the need for cautious and critical thinking about how to tailor PSMA-TRT approaches to individual patient characteristics and disease trajectories, ultimately aiming to optimize treatment efficacy and minimize adverse effects.

Given its recent introduction, long-term data on PSMA-TRT outcomes remain unavailable. However, short-term observations indicate that PSMA-targeted radionuclide therapy (PSMA-TRT) indeed presents side effects, both long- and short-term, respectively, to the patient. Commonly reported short-term effects include fatigue, nausea, and pain flares, typically resolving within one week post treatment [8]. Additionally, PSMA-TRT may induce xerostomia (dry mouth) and myelosuppression (a reduction in red blood cells) and result in mild radioactivity exposure, particularly to individuals in close proximity to the patient post treatment [8].

Ongoing research endeavors therefore aim to optimize dosing regimens, improve patient selection criteria, and explore potential synergies with other therapeutic modalities, thereby advancing a more personalized and effective approach to managing prostate cancer.

## 4. Understanding the Dynamics of PSMA-Based Treatments in Prostate Cancer Care

Dr. Scott T. Tagawa emphasized both the favorable and unfavorable outcomes associated with PSMA-based treatments. PSMA serves as an effective imaging biomarker, offering the potential to significantly improve the therapeutic ratio through pre-selection. However, as its primary advantage lies in its ability to simultaneously assess various tumors, this may also lead to it finding intratumoral heterogeneities. Moreover, considerations regarding the biodistribution and kinetics of drug development are paramount in this context.

Despite notable successes, PSMA-based assessments have some limitations. These evaluations are sequential and heavily reliant on target size and expression, often serving as a last option in challenging cases. Additionally, TRT has unmet needs, particularly regarding posology; therefore, the appropriate dosage of medications based on factors such as age, weight, and medical conditions, are required to ensure safe and effective treatment [17].

The outcomes of PSMA treatments are deeply dependent on two factors: the administered dose and PSMA’s uptake and retention, which influence both clinical and genomic outcomes. Moreover, different targeting agents, known as ‘keys’, present distinct characteristics. Antibodies, while effective for optimal imaging days later, may lead to predicted side effects such as infusion reactions and off-tumor exposure. In contrast, ligands, smaller in size, offer rapid tissue penetration but enhanced risks to various organs, including the kidney, salivary glands, lacrimal glands, and small intestine. In addition, it is crucial to note that PSMA binding sites are non-competitive across cell lines and exhibit additive binding. Case reports of ac-PSMA-617 highlight the dose-dependent variations in outcomes, reflecting the global interest and research in PSMA-based therapies.

Lastly, targeted alpha emitters, a subset of PSMA therapies, typically present a single-dose regimen. Careful counseling on the clinical implications of PSMA, supported by comprehensive assessments and interpretations, is therefore fundamental. Further research is essential to understand the efficacy and mechanisms of PSMA-based treatments, highlighting the importance of rigorous data collection and analysis. While existing studies provide insights into PSMA’s potential, ongoing research endeavors are pivotal to determine its efficacy and optimize its clinical utility.

## 5. Advancements in PSMA-Based Cancer Therapies: New promising Options and Combinations

It is important to recognize that patients undergoing PSMA treatment often receive other concurrent therapies to manage their cancer. By examining how PSMA interacts with these additional treatments, we can better understand how to optimize patient care. Notably, research on PSMA in combination with immunotherapy, initially tested in mice and later in human clinical trials, demonstrated a significant reduction in PSA levels in 76% of patients [18].

Several ongoing studies are exploring alternative combination therapies involving PSMA [8]. One such investigation involves combining PSMA with radionuclide therapy (RNT), alongside external beam radiation or brachytherapy. This approach aims to target and treat PSMA-expressing cells, potentially shortening the duration of radiation exposure in the body. While these studies are still in their early stages, they appear promising for enhancing prostate cancer treatment outcomes [19].

Additionally, current research is aiming to evaluate the efficacy of combining PSMA with radiosensitizers for prostate cancer treatment. Early findings suggest that DNA-damaging pharmaceuticals like olaparib and rucaparib, when used in combination with PSMA, may offer an effective treatment strategy [20]. Continued encouraging research in this area could lead to improved patient survival rates, particularly in cases of metastatic cancer.

The use of PSMA as a targeting agent could lead to a significantly more precise treatment by facilitating accurate cancer localization and potentially reducing recurrence rates. However, further investigation is needed to validate the clinical utility and relevance of combination therapies involving PSMA. Given the relatively recent introduction of PSMA-based treatments, ongoing monitoring of their long-term effects is essential.

Numerous combination therapies involving PSMA are currently undergoing phase I and phase II clinical testing, to enhance treatment efficacy and establish PSMA-targeted radionuclide therapy (PSMA-TRT) as a standard-of-care option. As more clinical trials are completed, we anticipate significant advancements in patient care and treatment outcomes.

Beyond prostate cancer, there is growing interest in the potential applications of PSMA-TRT to other cancer types. Preliminary studies have shown promising results in the detection and treatment of various malignancies, including salivary gland cancer, thyroid cancer, hepatocellular carcinoma, renal cell carcinoma, glioblastoma, breast cancer, and lung cancer, among others [11]. While further research is needed to confirm these findings, the potential of PSMA in diagnosing and treating cancer offers hope for improved outcomes and survival rates among several types of cancer patients.

In conclusion, the evolving landscape of PSMA-based therapies has the potential to redefine oncology treatment, offering new options for personalized and effective cancer management. Continued research and clinical trials are essential to exploit the full potential of PSMA in improving patient outcomes across various cancer types.

## 6. Conclusions and Future Perspectives

As discussed by Dr. Scott T. Tagawa, PSMA therapy represents a groundbreaking addition to clinical practice, although we must acknowledge that its journey toward maturity is still underway. Given the novel nature of PSMA therapy, its long-term complications are still unknown, but the efficacy of PSMA therapies outweighs their associated risks. Current and proposed treatments have significantly improved patient survival rates and quality of life, and further research could further enhance patient outcomes.

PSMA has emerged as a clinically validated and consistently relevant therapeutic target in the medical landscape. Dose–response data testify to its effectiveness in treating prostate cancer, a radiosensitive malignancy. This precision allows for the delivery of high doses of radiation to tumors while sparing adjacent healthy tissues. Such advancements pave the way for the development of novel combination therapies aimed at full cancer remission. Moreover, PSMA’s presence on various solid tumors beyond prostate cancer raises hopes for its utility in identifying and treating other carcinomas.

The success and widespread adoption of PSMA-targeted therapy have therefore sparked interest in the potential use of biomarkers to delineate and target cancers at various stages. Other biomarkers, such as Alpha-fetoprotein (AFP) in liver cancer, ovarian cancer, and germ cell tumors or BCL2 gene rearrangement as a biomarker in lymphomas and leukemia, offer additional options for diagnosis and distinguishing between the cancer presented, much like PSMA [21]. In particular, the tumor biomarker AFP is used to specifically help diagnose these cancers and determine their stage, prognosis, and the best approach for treatment [21]. Without these critical biomarkers, it could be significantly harder for providers to diagnose a specific cancer, especially if it has metastasized. Antibody–drug conjugates currently stand as the most commonly employed treatment modality, with theranostics being heralded as a promising frontier for advancing cancer therapeutics.

In summary, PSMA therapy represents a transformative era in cancer treatment. While its challenges persist, ongoing research and innovation have the potential to improve patient outcomes across diverse cancer types.

## Data Availability

No patient data were directly utilized in this study.

## References

[1] World Cancer Research Fund International Prostate Cancer Statistics. https://www.wcrf.org/cancer-trends/prostate-cancer-statistics/.

[2] ASCO Prostate Cancer—Statistics. https://www.cancer.net/cancer-types/prostate-cancer/statistics#:~:text=Prostate%20cancer%20is%20the%20second,worldwide%20died%20from%20prostate%20cancer.

[3] Sun M., Niaz M.J., Niaz M.O., Tagawa S.T. (2021). Prostate-Specific Membrane Antigen (PSMA)-Targeted Radionuclide Therapies for Prostate Cancer. Curr. Oncol. Rep..

[4] Chang S.S. (2004). Overview of prostate-specific membrane antigen. Rev. Urol..

[5] MedNews. Week MedNews Week Keynote Conference—Dr. Scott T. Tagawa (Weill Cornell Medicine)—Prostate Cancer. YouTube 2023. https://www.youtube.com/watch?v=DxHoNF3IyJI&list=PL2zMlh_ryhUzA6ozGZC-7QQmRqvWqqKH4&index=1.

[6] He M., Cao Y., Chi C., Zhao J., Chong E., Chin K.X.C., Tan N.Z.V., Dmitry K., Yang G., Yang X. (2023). Unleashing Novel Horizons in Advanced Prostate Cancer Treatment: Investigating the Potential of Prostate Specific Membrane Antigen-Targeted Nanomedicine-Based Combination Therapy. Front. Immunol..

[7] Malik A., Srinivasan S., Batra J. (2019). A New Era of Prostate Cancer Precision Medicine. Front. Oncol..

[8] Tagawa S. Transforming care for patients with prostate cancer by targeting prostate-specific membrane antigen. Proceedings of the MedNews Week Keynote Conference.

[9] Miyahira A.K., Soule H.R. (2021). The History of Prostate-Specific Membrane Antigen as a Theranostic Target in Prostate Cancer: The Cornerstone Role of the Prostate Cancer Foundation. J. Nucl. Med..

[10] Plichta K.A., Graves S.A., Buatti J.M. (2021). Prostate-Specific Membrane Antigen (PSMA) Theranostics for Treatment of Oligometastatic Prostate Cancer. Int. J. Mol. Sci..

[11] Lauri C., Chiurchioni L., Russo V.M., Zannini L., Signore A. (2022). PSMA Expression in Solid Tumors beyond the Prostate Gland: Ready for Theranostic Applications?. J. Clin. Med..

[12] Debnath S., Zhou N., McLaughlin M., Rice S., Pillai A.K., Hao G., Sun X. (2022). PSMA-Targeting Imaging and Theranostic Agents—Current Status and Future Perspective. Int. J. Mol. Sci..

[13] Werner R.A., Derlin T., Lapa C., Sheikbahaei S., Higuchi T., Giesel F.L., Behr S., Drzezga A., Kimura H., Buck A.K. (2020). 18F-Labeled, PSMA-Targeted Radiotracers: Leveraging the Advantages of Radiofluorination for Prostate Cancer Molecular Imaging. Theranostics.

[14] Echavidre W., Fagret D., Faraggi M., Picco V., Montemagno C. (2023). Recent Pre-Clinical Advancements in Nuclear Medicine: Pioneering the Path to a Limitless Future. Cancers.

[15] Pollack A., Zagars G.K., Kopplin S. (1995). Radiotherapy and androgen ablation for clinically localized high-risk prostate cancer. Int. J. Radiat. Oncol. Biol. Phys..

[16] Jadvar H., Baum R.P. (2024). Precision Oncology with PSMA-Targeted α-Particle Therapy of mCRPC. Beyond Becquerel and Biology to Precision Radiomolecular Oncology: Festschrift in Honor of Richard P. Baum.

[17] ASCO Daily News. What to Do with a Positive PSMA PET and Negative Conventional Imaging in Patients with Prostate Cancer. https://dailynews.ascopubs.org/do/do-positive-psma-pet-and-negative-conventional-imaging-patients-prostate-cancer.

[18] Sandhu S., Joshua A.M., Emmett L., Spain L.A., Horvath L., Crumbaker M., Anton A., Wallace R., Pasam A., Bressel M. (2022). PRINCE: Phase I trial of 177Lu-PSMA-617 in combination with pembrolizumab in patients with metastatic castration-resistant prostate cancer (mCRPC). J. Clin. Oncol..

[19] Inderjeeth A.J., Iravani A., Subramaniam S., Conduit C., Sandhu S. (2023). Novel radionuclide therapy combinations in prostate cancer. Ther. Adv. Med. Oncol..

[20] Arbuznikova D., Eder M., Grosu A.-L., Meyer P.T., Gratzke C., Zamboglou C., Eder A.-C. (2023). Towards Improving the Efficacy of PSMA-Targeting Radionuclide Therapy for Late-Stage Prostate Cancer-Combination Strategies. Curr. Oncol. Rep..

[21] Tumor Marker Tests in Common Use. National Cancer Institute Website. https://www.cancer.gov/about-cancer/diagnosis-staging/diagnosis/tumor-markers-list.

